# Real-time uptake of fluorescent ASP+ via the organic cation transporter 3

**DOI:** 10.1186/2050-6511-13-S1-A80

**Published:** 2012-09-17

**Authors:** Felix P Mayer, Diethart Schmid, Marion Holy, Jae-Won Yang, Isabella Salzer, Stefan Boehm, Peter Chiba, Harald H Sitte

**Affiliations:** 1 Center for Physiology and Pharmacology, Medical University of Vienna, 1090 Vienna, Austria; 2 Institute of Medical Chemistry, Medical University of Vienna, 1090 Vienna, Austria

## Background

The organic cation transporter 3 (OCT3) shows a broad expression pattern in the nervous system and has been detected in glial cells and neurons. Here, OCT3 serves as an additional high-capacity, low-affinity reuptake system for monoamine neurotransmitters such as norepinephrine, serotonin and dopamine, and therefore OCT-mediated uptake has been termed “uptake 2”.

## Methods

We used the mouse and human isoforms of OCT3 and stably expressed them in HEK 293 cells. We measured OCT3-mediated uptake of the fluorescent substrate 4-Di-1-ASP (4-(4-(dimethylamino)styryl)-*N*-methylpyridinium (ASP+) in real-time. Uptake of tritiated 1-methyl-4-phenylpyridinium was measured in comparison to the fluorescent uptake measurements. We used mass spectrometry to assess the phosphorylation status of OCT3.

## Results

We show that ASP+ is selectively taken up via OCT3 in real time. ASP+ uptake allows for sensitive assessment of transport via OCT3 and hence, we exploited the mode of action of several OCT3 substrates and transport inhibitors such as the stress hormone corticosterone. All results with the fluorescent ASP+ are in line with previously published reports. Finally, we tested if OCT-mediated uptake is sensitive to phosphorylation and observed that GF109203X inhibited uptake.

## Conclusions

Uptake by OCT3 can be measured with the fluorescent ligand ASP+; this uptake is comparable to the uptake of radioactively labeled MPP+. Uptake inhibition by corticosterone was comparable using either ASP+ or MPP+ and similar to inhibition of protein kinase C.

